# *N*-[4-(4-Nitro­phen­oxy)phen­yl]acetamide

**DOI:** 10.1107/S1600536812031856

**Published:** 2012-07-18

**Authors:** Asifa Nigar, Zareen Akhter, M. Nawaz Tahir

**Affiliations:** a Department of Chemistry, Quaid-i-Azam University, Islamabad, Pakistan; b Department of Physics, University of Sargodha, Sargodha, Pakistan

## Abstract

The asymmetric unit of the title compound, C_14_H_12_N_2_O_4_, contains two mol­ecules that differ principally in the orientation of the acetamide substituent to the adjacent benzene ring with dihedral angles of 44.77 (7) and 19.06 (7)°. The dihedral angles between the benzene rings are 64.46 (4) and 80.84 (4)°. In the crystal, classical N—H⋯O hydrogen bonds form *C*(4) chains along [100]. These chains are inter­linked by C—H⋯O contacts forming *R*_2_^2^(10) rings. In the crystal, π–π inter­actions are observed with a distance of 3.5976 (18) Å between the centroids of the nitro-substituted benzene rings of one type of mol­ecule.

## Related literature

For a related structure, see: Nigar *et al.* (2008 ▶). For hydrogen-bond motifs, see: Bernstein *et al.* (1995 ▶).
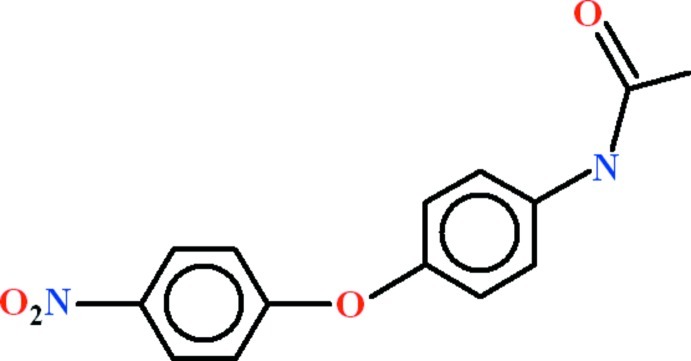


## Experimental

### 

#### Crystal data

C_14_H_12_N_2_O_4_
*M* *_r_* = 272.26
Triclinic, 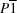

*a* = 9.6761 (6) Å
*b* = 10.4865 (7) Å
*c* = 14.3805 (14) Å
α = 98.779 (4)°
β = 98.641 (4)°
γ = 109.681 (3)°
*V* = 1325.57 (18) Å^3^
*Z* = 4
Mo *K*α radiation
μ = 0.10 mm^−1^
*T* = 296 K
0.35 × 0.28 × 0.24 mm

#### Data collection

Bruker Kappa APEXII CCD diffractometer
Absorption correction: multi-scan (*SADABS*; Bruker, 2005 ▶) *T*_min_ = 0.948, *T*_max_ = 0.968
21059 measured reflections
5876 independent reflections
4294 reflections with *I* > 2σ(*I*)
*R*_int_ = 0.020

#### Refinement

*R*[*F* ^2^ > 2σ(*F* ^2^)] = 0.041
*wR*(*F* ^2^) = 0.118
*S* = 1.03
5876 reflections
363 parameters
H-atom parameters constrained
Δρ_max_ = 0.18 e Å^−3^
Δρ_min_ = −0.18 e Å^−3^

### 

Data collection: *APEX2* (Bruker, 2009 ▶); cell refinement: *SAINT* (Bruker, 2009 ▶); data reduction: *SAINT*; program(s) used to solve structure: *SHELXS97* (Sheldrick, 2008 ▶); program(s) used to refine structure: *SHELXL97* (Sheldrick, 2008 ▶); molecular graphics: *ORTEP-3 for Windows* (Farrugia, 1997 ▶) and *PLATON* (Spek, 2009 ▶); software used to prepare material for publication: *WinGX* (Farrugia, 1999 ▶) and *PLATON*.

## Supplementary Material

Crystal structure: contains datablock(s) global, I. DOI: 10.1107/S1600536812031856/sj5257sup1.cif

Structure factors: contains datablock(s) I. DOI: 10.1107/S1600536812031856/sj5257Isup2.hkl

Supplementary material file. DOI: 10.1107/S1600536812031856/sj5257Isup3.cml

Additional supplementary materials:  crystallographic information; 3D view; checkCIF report

## Figures and Tables

**Table 1:** Hydrogen-bond geometry (Å, °)

*D*—H⋯*A*	*D*—H	H⋯*A*	*D*⋯*A*	*D*—H⋯*A*
N1—H1⋯O5^i^	0.86	2.04	2.8897 (19)	170
N3—H3⋯O1^ii^	0.86	2.10	2.941 (2)	165
C11—H11⋯O4^iii^	0.93	2.58	3.395 (2)	146

Symmetry codes: (i) 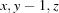; (ii) 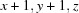; (iii) 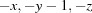.

**Fig. 1.**
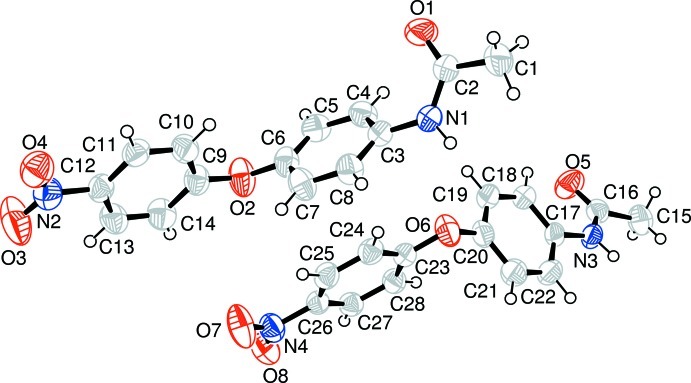
View of the title compound with displacement ellipsoids are drawn at the 50% probability level.

**Fig. 2.**
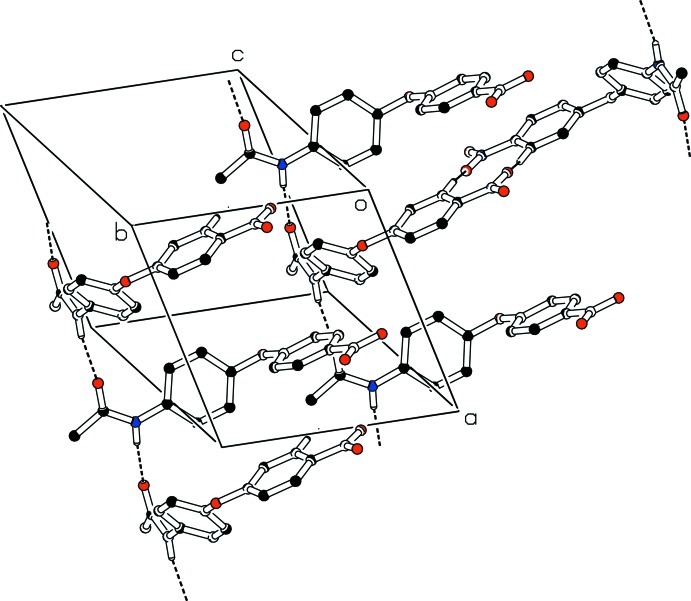
A partial packing diagram (PLATON; Spek, 2009) which shows that molecules form C(4) chains extending along the a-axis. Hydrogen bonds are drawn as dashed lines.

## supplementary crystallographic information

### Comment

The crystal structures of *N*-(4-(4-nitrophenoxy)phenyl)propionamide (Nigar *et al.* 2008) has been published and is related to that of (I), (Fig. 1).

In (I), two molecules (M1 and M2) are present in the asymmetric unit, which differ slightly from each other geometrically. In molecule M1, the acetamide group A (C1/C2/N1/O1), benzene ring B (C3—C8) and group C (O2/C9—C14/N2/O3/O4) of the 4-nitrophenol are almost planar with r.m.s. deviations of 0.0035 Å, 0.0109 Å and 0.0540 Å, respectively. The dihedral angles between A/B, A/C and B/C are 44.77 (7)°, 77.53 (7)° and 64.46 (4)°, respectively. In the second molecule M2, the comparable groups D (C15/C16/N3/O5), E (C17—C22) and F (O6/C23—C28/N4/O7/O8) are again almost planar with r.m.s. deviations of 0.0062 Å, 0.0031 Å and 0.0137 Å, respectively. The dihedral angles between D/E, D/F and E/F are 19.06 (7)°, 80.26 (5)° and 80.84 (4)°, respectively. Both molecules are interlinked forming C (4) chains (Bernstein *et al.*, 1995) due to classical N—H···O H–bonds (Table 1, Fig. 2). These infinite one-dimensional chains form along [100] and are further interlinked through C–H···O contacts forming *R*_2_^2^(10) rings (Table 1, Fig. 2). A π–π interaction is also present, *Cg*1···*Cg*1^i^ [i = 1 - *x*, 1 - *y*, - *z*] at a distance of 3.8814 (10) Å, where *Cg*1 is the centroid of the (C23—C28) benzene ring .

### Experimental

In the first step 4-(4-nitrophenoxy)aniline was prepared from a mixture of 4-aminophenol (5.046 g, 50 mmol), 4-nitrofluorobenzene (5.3 ml, 50 mmol) and anhydrous K_2_CO_3_ (6.91 g, 50 mmol) in 70 ml dimethylformamide (DMF) by heating and stirring at 373 K for 18 h in an inert atmosphere. After cooling to room temperature, the reaction mixture was poured into 800 ml of water to yield a yellow solid. The product was filtered, dried, and then recrystallized from n-hexane (86% yield).

In the second step, acetylchloride was reacted with 4-(4-nitrophenoxy)aniline, in appropriate molar ratios in tetrahydrofuran with 1 ml of triethylamine for 1.0 g of 4-(4-nitrophenoxy)aniline. The reaction mixture was refluxed for 2 h under inert conditions and allowed to stand overnight at room temperature. The settled salt was filtered off and filterate was evaporated to get the crude product, which was recrystallized from toluene (92% yield, m.p. 428 K).

### Refinement

The H-atoms were positioned geometrically (C—H = 0.93–0.96 Å, N—H = 0.86 Å) and refined as riding with *U*_iso_(H) = *xU*_eq_(C, N), where *x* = 1.5 for methyl groups and *x* = 1.2 for other H atoms.

### Crystal data

**Table:**

C_14_H_12_N_2_O_4_	*Z* = 4
*M**_r_* = 272.26	*F*(000) = 568
Triclinic, *P*1	*D*_x_ = 1.364 Mg m^−^^3^
Hall symbol: -P 1	Mo *K*α radiation, λ = 0.71073 Å
*a* = 9.6761 (6) Å	Cell parameters from 4294 reflections
*b* = 10.4865 (7) Å	θ = 1.5–27.3°
*c* = 14.3805 (14) Å	µ = 0.10 mm^−^^1^
α = 98.779 (4)°	*T* = 296 K
β = 98.641 (4)°	Prism, yellow
γ = 109.681 (3)°	0.35 × 0.28 × 0.24 mm
*V* = 1325.57 (18) Å^3^	

### Data collection

**Table:**

Bruker Kappa APEXII CCD diffractometer	5876 independent reflections
Radiation source: fine-focus sealed tube	4294 reflections with *I* > 2σ(*I*)
Graphite monochromator	*R*_int_ = 0.020
Detector resolution: 7.80 pixels mm^-1^	θ_max_ = 27.3°, θ_min_ = 1.5°
ω scans	*h* = −12→12
Absorption correction: multi-scan (*SADABS*; Bruker, 2005)	*k* = −13→13
*T*_min_ = 0.948, *T*_max_ = 0.968	*l* = −18→18
21059 measured reflections	

### Refinement

**Table:**

Refinement on *F*^2^	Primary atom site location: structure-invariant direct methods
Least-squares matrix: full	Secondary atom site location: difference Fourier map
*R*[*F*^2^ > 2σ(*F*^2^)] = 0.041	Hydrogen site location: inferred from neighbouring sites
*wR*(*F*^2^) = 0.118	H-atom parameters constrained
*S* = 1.03	*w* = 1/[σ^2^(*F*_o_^2^) + (0.0524*P*)^2^ + 0.2302*P*] where *P* = (*F*_o_^2^ + 2*F*_c_^2^)/3
5876 reflections	(Δ/σ)_max_ < 0.001
363 parameters	Δρ_max_ = 0.18 e Å^−^^3^
0 restraints	Δρ_min_ = −0.18 e Å^−^^3^

### Special details

**Table:**

Geometry. Bond distances, angles *etc*. have been calculated using the rounded fractional coordinates. All su's are estimated from the variances of the (full) variance-covariance matrix. The cell e.s.d.'s are taken into account in the estimation of distances, angles and torsion angles
Refinement. Refinement of *F*^2^ against ALL reflections. The weighted *R*-factor *wR* and goodness of fit *S* are based on *F*^2^, conventional *R*-factors *R* are based on *F*, with *F* set to zero for negative *F*^2^. The threshold expression of *F*^2^ > σ(*F*^2^) is used only for calculating *R*-factors(gt) *etc*. and is not relevant to the choice of reflections for refinement. *R*-factors based on *F*^2^ are statistically about twice as large as those based on *F*, and *R*- factors based on ALL data will be even larger.

### Fractional atomic coordinates and isotropic or equivalent isotropic displacement parameters (Å^2^)

**Table:**

	*x*	*y*	*z*	*U*_iso_*/*U*_eq_	
O1	0.40441 (14)	0.21132 (19)	0.49456 (9)	0.0958 (6)	
O2	0.25849 (15)	0.10057 (11)	0.04136 (8)	0.0722 (4)	
O3	−0.0660 (2)	−0.44739 (16)	−0.27043 (10)	0.1154 (7)	
O4	−0.06874 (14)	−0.53295 (13)	−0.14489 (9)	0.0755 (5)	
N1	0.59193 (13)	0.22220 (13)	0.41723 (8)	0.0546 (4)	
N2	−0.03678 (16)	−0.43416 (15)	−0.18388 (10)	0.0649 (5)	
C1	0.6417 (2)	0.2424 (3)	0.58930 (12)	0.0826 (7)	
C2	0.53480 (17)	0.22473 (18)	0.49691 (11)	0.0604 (5)	
C3	0.50643 (15)	0.19218 (14)	0.32193 (10)	0.0475 (4)	
C4	0.40583 (17)	0.25561 (16)	0.29751 (11)	0.0555 (5)	
C5	0.32052 (18)	0.22013 (17)	0.20475 (11)	0.0568 (5)	
C6	0.33923 (18)	0.12516 (15)	0.13617 (10)	0.0544 (5)	
C7	0.4426 (2)	0.06483 (16)	0.15806 (11)	0.0610 (5)	
C8	0.52464 (18)	0.09740 (15)	0.25154 (11)	0.0552 (5)	
C9	0.18649 (17)	−0.03352 (16)	−0.01071 (10)	0.0544 (5)	
C10	0.13163 (18)	−0.14559 (16)	0.03176 (11)	0.0583 (5)	
C11	0.05864 (16)	−0.27668 (16)	−0.02517 (11)	0.0535 (5)	
C12	0.03935 (16)	−0.29410 (15)	−0.12364 (10)	0.0510 (5)	
C13	0.09235 (19)	−0.18353 (17)	−0.16698 (11)	0.0602 (5)	
C14	0.16669 (19)	−0.05321 (17)	−0.10996 (11)	0.0619 (6)	
O5	0.90148 (13)	1.24924 (13)	0.42809 (10)	0.0759 (5)	
O6	0.78524 (14)	0.60256 (11)	0.25638 (7)	0.0653 (4)	
O7	0.52827 (19)	0.23177 (14)	−0.15684 (10)	0.0963 (6)	
O8	0.59239 (15)	0.43167 (14)	−0.19322 (8)	0.0774 (5)	
N3	1.08670 (13)	1.16492 (12)	0.42198 (8)	0.0492 (4)	
N4	0.58274 (16)	0.35854 (15)	−0.13432 (10)	0.0612 (5)	
C15	1.1524 (2)	1.40466 (17)	0.49924 (12)	0.0678 (6)	
C16	1.03475 (17)	1.26684 (15)	0.44778 (10)	0.0512 (5)	
C17	1.00375 (15)	1.02506 (14)	0.37582 (9)	0.0444 (4)	
C18	0.85180 (16)	0.95950 (16)	0.37204 (10)	0.0532 (5)	
C19	0.77942 (17)	0.82020 (16)	0.32895 (11)	0.0570 (5)	
C20	0.85795 (18)	0.74671 (15)	0.29088 (10)	0.0527 (5)	
C21	1.00941 (19)	0.80898 (16)	0.29404 (12)	0.0603 (5)	
C22	1.08123 (17)	0.94831 (16)	0.33607 (12)	0.0574 (5)	
C23	0.73666 (15)	0.55009 (14)	0.15953 (10)	0.0466 (4)	
C24	0.67086 (16)	0.40675 (15)	0.13192 (11)	0.0513 (5)	
C25	0.62091 (16)	0.34379 (15)	0.03588 (11)	0.0525 (5)	
C26	0.63580 (15)	0.42461 (14)	−0.03208 (10)	0.0467 (4)	
C27	0.69734 (16)	0.56715 (15)	−0.00523 (10)	0.0486 (5)	
C28	0.74872 (16)	0.63090 (14)	0.09119 (10)	0.0487 (4)	
H1	0.68689	0.24002	0.42456	0.0654*	
H1A	0.64663	0.32191	0.63473	0.1239*	
H1B	0.73978	0.25551	0.57700	0.1239*	
H1C	0.60693	0.16108	0.61528	0.1239*	
H4	0.39564	0.32234	0.34370	0.0667*	
H5	0.25069	0.26063	0.18900	0.0681*	
H7	0.45707	0.00281	0.11051	0.0732*	
H8	0.59277	0.05510	0.26719	0.0662*	
H10	0.14429	−0.13190	0.09840	0.0700*	
H11	0.02275	−0.35261	0.00249	0.0641*	
H13	0.07777	−0.19741	−0.23371	0.0722*	
H14	0.20391	0.02215	−0.13793	0.0743*	
H3	1.18280	1.18853	0.43550	0.0590*	
H15A	1.16040	1.46941	0.45792	0.1016*	
H15B	1.24735	1.39449	0.51602	0.1016*	
H15C	1.12472	1.43840	0.55674	0.1016*	
H18	0.79841	1.00930	0.39854	0.0639*	
H19	0.67724	0.77662	0.32593	0.0684*	
H21	1.06219	0.75794	0.26829	0.0724*	
H22	1.18305	0.99153	0.33782	0.0689*	
H24	0.66057	0.35339	0.17833	0.0616*	
H25	0.57745	0.24760	0.01674	0.0630*	
H27	0.70426	0.62020	−0.05181	0.0584*	
H28	0.79103	0.72716	0.11014	0.0585*	

### Atomic displacement parameters (Å^2^)

**Table:**

	*U*^11^	*U*^22^	*U*^33^	*U*^12^	*U*^13^	*U*^23^
O1	0.0473 (7)	0.1819 (15)	0.0574 (7)	0.0411 (8)	0.0140 (5)	0.0239 (8)
O2	0.0933 (9)	0.0528 (6)	0.0546 (6)	0.0168 (6)	−0.0108 (6)	0.0156 (5)
O3	0.1624 (16)	0.0864 (10)	0.0554 (8)	0.0135 (10)	−0.0088 (9)	0.0002 (7)
O4	0.0742 (8)	0.0594 (7)	0.0871 (9)	0.0194 (6)	0.0144 (7)	0.0139 (6)
N1	0.0380 (6)	0.0712 (8)	0.0487 (7)	0.0171 (6)	0.0060 (5)	0.0066 (6)
N2	0.0606 (8)	0.0653 (9)	0.0617 (9)	0.0204 (7)	0.0047 (7)	0.0082 (7)
C1	0.0612 (11)	0.1266 (17)	0.0519 (9)	0.0329 (11)	0.0014 (8)	0.0108 (10)
C2	0.0436 (8)	0.0809 (11)	0.0484 (8)	0.0170 (8)	0.0061 (7)	0.0073 (8)
C3	0.0410 (7)	0.0504 (8)	0.0468 (7)	0.0122 (6)	0.0089 (6)	0.0094 (6)
C4	0.0577 (9)	0.0621 (9)	0.0526 (8)	0.0273 (8)	0.0166 (7)	0.0126 (7)
C5	0.0548 (9)	0.0646 (9)	0.0591 (9)	0.0278 (8)	0.0130 (7)	0.0231 (8)
C6	0.0580 (9)	0.0478 (8)	0.0486 (8)	0.0110 (7)	0.0007 (7)	0.0149 (7)
C7	0.0779 (11)	0.0496 (8)	0.0527 (9)	0.0259 (8)	0.0072 (8)	0.0045 (7)
C8	0.0562 (9)	0.0536 (8)	0.0558 (9)	0.0247 (7)	0.0050 (7)	0.0090 (7)
C9	0.0545 (9)	0.0540 (9)	0.0493 (8)	0.0170 (7)	0.0001 (7)	0.0137 (7)
C10	0.0608 (10)	0.0650 (10)	0.0431 (8)	0.0151 (8)	0.0073 (7)	0.0168 (7)
C11	0.0459 (8)	0.0580 (9)	0.0533 (8)	0.0125 (7)	0.0095 (6)	0.0194 (7)
C12	0.0423 (8)	0.0562 (9)	0.0508 (8)	0.0179 (7)	0.0025 (6)	0.0093 (7)
C13	0.0682 (10)	0.0682 (10)	0.0430 (8)	0.0251 (8)	0.0047 (7)	0.0159 (7)
C14	0.0723 (11)	0.0612 (10)	0.0515 (9)	0.0210 (8)	0.0074 (8)	0.0241 (7)
O5	0.0526 (7)	0.0741 (8)	0.1034 (10)	0.0312 (6)	0.0142 (6)	0.0105 (7)
O6	0.0835 (8)	0.0496 (6)	0.0447 (6)	0.0054 (5)	0.0059 (5)	0.0096 (4)
O7	0.1340 (13)	0.0609 (8)	0.0720 (9)	0.0342 (8)	−0.0108 (8)	−0.0125 (6)
O8	0.0944 (10)	0.0862 (9)	0.0503 (7)	0.0364 (7)	0.0099 (6)	0.0095 (6)
N3	0.0372 (6)	0.0520 (7)	0.0519 (7)	0.0148 (5)	0.0011 (5)	0.0048 (5)
N4	0.0621 (8)	0.0636 (9)	0.0554 (8)	0.0297 (7)	0.0043 (6)	−0.0010 (7)
C15	0.0764 (12)	0.0556 (9)	0.0631 (10)	0.0231 (8)	0.0035 (8)	0.0035 (8)
C16	0.0535 (9)	0.0572 (9)	0.0438 (7)	0.0224 (7)	0.0084 (6)	0.0107 (6)
C17	0.0414 (7)	0.0504 (8)	0.0369 (7)	0.0148 (6)	0.0010 (5)	0.0085 (6)
C18	0.0439 (8)	0.0608 (9)	0.0509 (8)	0.0169 (7)	0.0087 (6)	0.0075 (7)
C19	0.0442 (8)	0.0639 (10)	0.0510 (8)	0.0074 (7)	0.0071 (7)	0.0103 (7)
C20	0.0580 (9)	0.0485 (8)	0.0391 (7)	0.0075 (7)	0.0037 (6)	0.0080 (6)
C21	0.0593 (10)	0.0563 (9)	0.0631 (9)	0.0227 (8)	0.0125 (8)	0.0039 (7)
C22	0.0423 (8)	0.0570 (9)	0.0669 (10)	0.0150 (7)	0.0101 (7)	0.0056 (7)
C23	0.0414 (7)	0.0478 (8)	0.0455 (7)	0.0112 (6)	0.0089 (6)	0.0082 (6)
C24	0.0490 (8)	0.0467 (8)	0.0556 (8)	0.0121 (6)	0.0121 (7)	0.0154 (6)
C25	0.0479 (8)	0.0415 (7)	0.0619 (9)	0.0127 (6)	0.0094 (7)	0.0047 (7)
C26	0.0399 (7)	0.0513 (8)	0.0470 (7)	0.0187 (6)	0.0073 (6)	0.0028 (6)
C27	0.0485 (8)	0.0512 (8)	0.0479 (8)	0.0189 (7)	0.0118 (6)	0.0132 (6)
C28	0.0495 (8)	0.0411 (7)	0.0505 (8)	0.0115 (6)	0.0102 (6)	0.0079 (6)

### Geometric parameters (Å, º)

**Table:**

O1—C2	1.216 (2)	C1—H1B	0.9600
O2—C6	1.4045 (19)	C4—H4	0.9300
O2—C9	1.3731 (19)	C5—H5	0.9300
O3—N2	1.210 (2)	C7—H7	0.9300
O4—N2	1.224 (2)	C8—H8	0.9300
O5—C16	1.221 (2)	C10—H10	0.9300
O6—C20	1.4057 (19)	C11—H11	0.9300
O6—C23	1.3633 (17)	C13—H13	0.9300
O7—N4	1.223 (2)	C14—H14	0.9300
O8—N4	1.2199 (19)	C15—C16	1.498 (2)
N1—C3	1.4191 (18)	C17—C18	1.386 (2)
N1—C2	1.345 (2)	C17—C22	1.390 (2)
N2—C12	1.463 (2)	C18—C19	1.383 (2)
N1—H1	0.8600	C19—C20	1.364 (2)
N3—C17	1.4092 (18)	C20—C21	1.379 (3)
N3—C16	1.353 (2)	C21—C22	1.380 (2)
N4—C26	1.459 (2)	C23—C28	1.386 (2)
N3—H3	0.8600	C23—C24	1.386 (2)
C1—C2	1.501 (2)	C24—C25	1.372 (2)
C3—C4	1.385 (2)	C25—C26	1.381 (2)
C3—C8	1.381 (2)	C26—C27	1.376 (2)
C4—C5	1.382 (2)	C27—C28	1.379 (2)
C5—C6	1.368 (2)	C15—H15A	0.9600
C6—C7	1.376 (3)	C15—H15B	0.9600
C7—C8	1.382 (2)	C15—H15C	0.9600
C9—C14	1.385 (2)	C18—H18	0.9300
C9—C10	1.390 (2)	C19—H19	0.9300
C10—C11	1.374 (2)	C21—H21	0.9300
C11—C12	1.376 (2)	C22—H22	0.9300
C12—C13	1.383 (2)	C24—H24	0.9300
C13—C14	1.370 (2)	C25—H25	0.9300
C1—H1A	0.9600	C27—H27	0.9300
C1—H1C	0.9600	C28—H28	0.9300
			
C6—O2—C9	119.33 (12)	C9—C10—H10	120.00
C20—O6—C23	119.32 (11)	C12—C11—H11	120.00
C2—N1—C3	124.57 (14)	C10—C11—H11	120.00
O3—N2—O4	122.66 (16)	C14—C13—H13	121.00
O4—N2—C12	118.86 (13)	C12—C13—H13	121.00
O3—N2—C12	118.48 (15)	C13—C14—H14	120.00
C3—N1—H1	118.00	C9—C14—H14	120.00
C2—N1—H1	118.00	O5—C16—N3	122.44 (14)
C16—N3—C17	128.40 (14)	N3—C16—C15	115.48 (15)
O7—N4—O8	123.02 (14)	O5—C16—C15	122.05 (15)
O7—N4—C26	118.13 (14)	N3—C17—C22	117.60 (14)
O8—N4—C26	118.84 (14)	N3—C17—C18	123.48 (14)
C17—N3—H3	116.00	C18—C17—C22	118.86 (14)
C16—N3—H3	116.00	C17—C18—C19	120.14 (15)
N1—C2—C1	115.69 (16)	C18—C19—C20	119.99 (16)
O1—C2—C1	121.80 (16)	C19—C20—C21	121.17 (15)
O1—C2—N1	122.50 (15)	O6—C20—C21	119.88 (15)
N1—C3—C4	121.86 (13)	O6—C20—C19	118.67 (15)
C4—C3—C8	119.15 (14)	C20—C21—C22	118.84 (16)
N1—C3—C8	118.99 (14)	C17—C22—C21	121.01 (16)
C3—C4—C5	120.25 (15)	C24—C23—C28	120.64 (13)
C4—C5—C6	119.72 (16)	O6—C23—C24	115.37 (13)
O2—C6—C7	121.33 (14)	O6—C23—C28	123.99 (13)
C5—C6—C7	120.91 (14)	C23—C24—C25	119.79 (14)
O2—C6—C5	117.55 (15)	C24—C25—C26	119.38 (14)
C6—C7—C8	119.25 (15)	N4—C26—C27	119.03 (13)
C3—C8—C7	120.64 (16)	N4—C26—C25	119.74 (13)
C10—C9—C14	120.37 (15)	C25—C26—C27	121.22 (13)
O2—C9—C14	116.64 (14)	C26—C27—C28	119.61 (13)
O2—C9—C10	122.98 (13)	C23—C28—C27	119.33 (13)
C9—C10—C11	119.69 (14)	C16—C15—H15A	109.00
C10—C11—C12	119.11 (15)	C16—C15—H15B	109.00
C11—C12—C13	121.87 (14)	C16—C15—H15C	109.00
N2—C12—C13	119.23 (13)	H15A—C15—H15B	109.00
N2—C12—C11	118.88 (14)	H15A—C15—H15C	109.00
C12—C13—C14	118.88 (14)	H15B—C15—H15C	109.00
C9—C14—C13	120.08 (15)	C17—C18—H18	120.00
H1B—C1—H1C	110.00	C19—C18—H18	120.00
C2—C1—H1A	109.00	C18—C19—H19	120.00
C2—C1—H1C	109.00	C20—C19—H19	120.00
H1A—C1—H1B	109.00	C20—C21—H21	121.00
C2—C1—H1B	109.00	C22—C21—H21	121.00
H1A—C1—H1C	109.00	C17—C22—H22	120.00
C3—C4—H4	120.00	C21—C22—H22	119.00
C5—C4—H4	120.00	C23—C24—H24	120.00
C6—C5—H5	120.00	C25—C24—H24	120.00
C4—C5—H5	120.00	C24—C25—H25	120.00
C6—C7—H7	120.00	C26—C25—H25	120.00
C8—C7—H7	120.00	C26—C27—H27	120.00
C7—C8—H8	120.00	C28—C27—H27	120.00
C3—C8—H8	120.00	C23—C28—H28	120.00
C11—C10—H10	120.00	C27—C28—H28	120.00
			
C9—O2—C6—C5	−135.22 (17)	C5—C6—C7—C8	2.3 (3)
C9—O2—C6—C7	50.0 (2)	C6—C7—C8—C3	−1.7 (3)
C6—O2—C9—C10	29.0 (3)	O2—C9—C10—C11	178.98 (17)
C6—O2—C9—C14	−152.54 (17)	C14—C9—C10—C11	0.6 (3)
C20—O6—C23—C28	−2.7 (2)	O2—C9—C14—C13	−178.28 (17)
C20—O6—C23—C24	177.91 (15)	C10—C9—C14—C13	0.2 (3)
C23—O6—C20—C19	103.06 (17)	C9—C10—C11—C12	−0.9 (3)
C23—O6—C20—C21	−82.94 (19)	C10—C11—C12—C13	0.5 (3)
C2—N1—C3—C4	48.3 (2)	C10—C11—C12—N2	179.23 (16)
C3—N1—C2—C1	173.13 (17)	C11—C12—C13—C14	0.3 (3)
C2—N1—C3—C8	−132.00 (17)	N2—C12—C13—C14	−178.43 (17)
C3—N1—C2—O1	−5.7 (3)	C12—C13—C14—C9	−0.6 (3)
O3—N2—C12—C11	173.12 (18)	N3—C17—C18—C19	−177.22 (13)
O3—N2—C12—C13	−8.1 (3)	C22—C17—C18—C19	−0.3 (2)
O4—N2—C12—C11	−6.7 (2)	N3—C17—C22—C21	176.67 (14)
O4—N2—C12—C13	172.12 (17)	C18—C17—C22—C21	−0.5 (2)
C16—N3—C17—C18	−18.1 (2)	C17—C18—C19—C20	0.6 (2)
C17—N3—C16—O5	−4.1 (2)	C18—C19—C20—O6	173.63 (13)
C17—N3—C16—C15	177.95 (13)	C18—C19—C20—C21	−0.3 (2)
C16—N3—C17—C22	164.90 (14)	O6—C20—C21—C22	−174.28 (14)
O8—N4—C26—C27	−1.1 (2)	C19—C20—C21—C22	−0.4 (2)
O8—N4—C26—C25	177.67 (16)	C20—C21—C22—C17	0.8 (2)
O7—N4—C26—C25	−1.2 (2)	O6—C23—C24—C25	−178.77 (15)
O7—N4—C26—C27	−179.94 (18)	C28—C23—C24—C25	1.9 (2)
N1—C3—C8—C7	179.57 (15)	O6—C23—C28—C27	179.30 (15)
C4—C3—C8—C7	−0.7 (2)	C24—C23—C28—C27	−1.4 (2)
C8—C3—C4—C5	2.6 (2)	C23—C24—C25—C26	−0.6 (2)
N1—C3—C4—C5	−177.67 (15)	C24—C25—C26—N4	−179.88 (15)
C3—C4—C5—C6	−2.1 (3)	C24—C25—C26—C27	−1.2 (2)
C4—C5—C6—O2	−175.18 (15)	N4—C26—C27—C28	−179.63 (15)
C4—C5—C6—C7	−0.4 (3)	C25—C26—C27—C28	1.6 (2)
O2—C6—C7—C8	176.87 (15)	C26—C27—C28—C23	−0.4 (2)

### Hydrogen-bond geometry (Å, º)

**Table:**

*D*—H···*A*	*D*—H	H···*A*	*D*···*A*	*D*—H···*A*
N1—H1···O5^i^	0.86	2.04	2.8897 (19)	170
N3—H3···O1^ii^	0.86	2.10	2.941 (2)	165
C11—H11···O4^iii^	0.93	2.58	3.395 (2)	146

Symmetry codes: (i) *x*, *y*−1, *z*; (ii) *x*+1, *y*+1, *z*; (iii) −*x*, −*y*−1, −*z*.

## References

[bb1] Bernstein, J., Davis, R. E., Shimoni, L. & Chang, N.-L. (1995). *Angew. Chem. Int. Ed. Engl.* **34**, 1555–1573.

[bb2] Bruker (2005). *SADABS* Bruker AXS Inc., Madison, Wisconsin, USA.

[bb3] Bruker (2009). *APEX2* and *SAINT* Bruker AXS Inc., Madison, Wisconsin, USA.

[bb4] Farrugia, L. J. (1997). *J. Appl. Cryst.* **30**, 565.

[bb5] Farrugia, L. J. (1999). *J. Appl. Cryst.* **32**, 837–838.

[bb6] Nigar, A., Akhter, Z., Bolte, M., Siddiqi, H. M. & Hussain, R. (2008). *Acta Cryst.* E**64**, o2186. 10.1107/S1600536808034119 PMC2959666 21581044

[bb7] Sheldrick, G. M. (2008). *Acta Cryst.* A**64**, 112–122. 10.1107/S0108767307043930 18156677

[bb8] Spek, A. L. (2009). *Acta Cryst.* D**65**, 148–155. 10.1107/S090744490804362X PMC2631630 19171970

